# From loneliness to internet addiction in adolescents: disentangling the within-person mediating role of rumination via RI-CLPM

**DOI:** 10.3389/fpubh.2026.1908127

**Published:** 2026-08-24

**Authors:** Yu Deng, Tianna Xiong, Baojuan Ye, Qi Dai, Kai Jing, Haibin Huang

**Affiliations:** 1 School of Education, Jiangxi Normal University, Nanchang, China; 2 Student Affairs Office, Zhaotong University, Zhaotong, China; 3 School of Psychology, Jiangxi Normal University, Nanchang, China; 4 Research Center for Positive Psychological Development, Jiangxi Normal University, Nanchang, China; 5 Student Affairs Office, Jiangxi Vocational College of Foreign Studies, Nanchang, China; 6 School of Medical Humanities and Management, Hunan University of Medicine, Huaihua, China

**Keywords:** adolescents, internet addiction, loneliness, random intercept cross-lagged panel model(RI-CLPM), rumination

## Abstract

**Background:**

Internet addiction symptoms have received increasing research attention as a public health concern among adolescents, with well-documented negative impacts on psychological wellbeing and social functioning. Although loneliness has been identified as an important correlate and prospective risk factor for Internet addiction symptoms, the longitudinal mechanisms underlying this relationship remain underexplored. This three-wave longitudinal study examines the mediating role of rumination in the link between loneliness and internet addiction among Chinese adolescents.

**Methods:**

A total of 743 Chinese junior high school students (*Mage* = 13.34, SD = 0.847; 51.7% female) completed measures of loneliness, rumination, and internet addiction at three time points spaced 6 months apart. A random intercept cross-lagged panel model (RI-CLPM) was employed to separate between-person trait variance from within-person temporal dynamics, allowing a more precise examination of reciprocal and mediational processes.

**Results:**

At the between-person level, the random intercepts of loneliness, rumination, and internet addiction were not significantly correlated. At the within-personmental health crisislevel, significant bidirectional and unidirectional cross-lagged effects emerged. Specifically, higher-than-usual loneliness predicted subsequent increases in rumination and internet addiction. Elevated rumination, in turn, predicted greater subsequent internet addiction. Internet addiction was also prospectively associated with subsequent increases in both loneliness and rumination, suggesting a reciprocal within-person cycle. Longitudinal mediation analyses revealed that rumination significantly mediated the within-person pathway from loneliness to self-reported Internet addiction. However, the standardized indirect effect was modest, suggesting limited practical magnitude. The reverse indirect effect was not significant.

**Conclusions:**

By employing RI-CLPM, this study delineates the dynamic within-person processes associated with adolescent internet addiction at the individual level. These findings identify rumination as a cognitive mechanism linking the emotional experience of loneliness to the development of Internet addiction symptoms. The results suggest the potential value of targeting both loneliness and maladaptive ruminative thinking in prevention and intervention efforts aimed at reducing internet addiction among adolescents.

## 1 Introduction

With the rapid advancement of internet technology, self-reported Internet addiction symptoms have received increasing research attention as a public health concern among adolescents (1, 2). Studies indicate that the prevalence of internet addiction in adolescents worldwide is approximately 10%, with rates exceeding 20% in some Asian regions, reflecting a trend toward broader and earlier onset (3). This behavioral issue is closely associated with a range of profound psychosocial impairments. Research has demonstrated that internet addiction is significantly linked to depression, anxiety, sleep disturbances, and notable declines in academic performance (4–8). Moreover, adolescents with internet addiction face a risk of social isolation and family conflict that is two to three times higher than their non-addicted peers, and they are more likely to be involved in cyberbullying and aggression. These interrelated problems further exacerbate psychological difficulties among adolescents (9, 10). Therefore, an in-depth investigation into internet addiction holds may contribute to theoretical understanding and prevention efforts for safeguarding and enhancing the mental health of young people.

In this study, Internet Addiction refers to maladaptive Internet use measured by self-report questionnaires, not a formal clinical diagnosis. In nonclinical research, Internet Addiction has been extensively studied using validated instruments such as Young's Internet Addiction Test (11), which provide dimensional assessments of excessive, poorly controlled Internet use. Although some researchers prefer the term “Problematic Internet Use” (PIU), recent taxonomic work indicates that Internet Addiction and PIU conceptually overlap in non-clinical populations, both reflecting functional impairment and loss of control (12, 13). Moreover, relying exclusively on application-specific terms (e.g., problematic gaming) may obscure transdiagnostic risk factors (14). Thus, Internet Addiction is used here as a continuous, dimensional construct measured by self-report scales, consistent with the literature on internet addiction and its psychosocial correlates.

### 1.1 The relationship between loneliness and internet addiction

The relationship between loneliness and internet addiction represents a central concern in the field of adolescent mental health. A substantial body of empirical research has established that loneliness is a important predictor of internet addiction (1, 2), a finding further corroborated by studies conducted during the COVID-19 pandemic (3). The Compensatory Internet Use Theory provides a principal framework for understanding this linkage. It posits that individuals experiencing heightened loneliness, often due to inadequate real-world social connections and a lack of belonging, may increasingly turn to the online environment. This shift serves to compensate for offline social deficits, escape psychological distress, and seek emotional support and relational fulfillment, thereby reinforcing dependence on the internet (4–7). Furthermore, the Deficient Self-regulation Model elucidates an additional pathway. It suggests that individuals with psychosocial difficulties frequently exhibit impaired self-regulatory capacity. This deficit undermines their ability to control online behavior, facilitating a progression toward addictive use patterns (8).

Conversely, the effect of internet addiction on exacerbating loneliness is also well-established. Excessive immersion in online activities can precipitate social displacement, leading to reduced participation in face-to-face interactions and a weakening of real-world relationships. This process is often conceptualized as a mediational pathway in which internet use leads to heightened loneliness via social displacement, thereby intensifying negative affective experiences (9). Critically, a growing consensus in the literature indicates a bidirectional, potentially cyclical relationship between these constructs. Evidence suggests that loneliness elevates the risk for internet addiction, which in turn may amplify feelings of loneliness, creating a self-perpetuating cycle (15, 16). However, this reciprocal relationship is highly complex and contingent upon various factors (16). Consequently, further research is needed to fully elucidate the dynamic and bidirectional nature of the interplay between loneliness and internet addiction.

### 1.2 The relationship between rumination and loneliness

Beyond the direct loneliness–internet addiction loop, a growing body of research suggests that a third variable—rumination—may serve as a plausible cognitive mechanism linking the two. Thus, we next examine the relationship between rumination and loneliness.

The close association between rumination and loneliness is supported by theoretical and empirical research. According to Nolen-Hoeksema's rumination theory (17), persistent reflection on negative events can trigger negative emotions and social avoidance (18). Meanwhile, social processing theory (19) suggests that rumination exacerbates loneliness when real-life interpersonal relationships fail to meet expectations by generating pessimistic expectations (20–22). Cross-sectional studies demonstrate that lonely individuals are susceptible to a vicious cycle in which loneliness leads to negative cognition, which in turn fosters rumination (23) and that loneliness in adolescents can directly trigger rumination tendencies (24). From a longitudinal perspective, the relationship between the two is not yet fully understood, but potential connections have emerged. It found that adolescents with higher levels of loneliness are more likely to become trapped in negative emotional rumination (25). However, other studies present an opposite pathway, indicating that surveys of university students show rumination as a positive predictor of loneliness (26). In summary, loneliness and rumination may be bidirectionally related, and further research is needed.

### 1.3 The relationship between rumination and internet addiction

Rumination is a maladaptive cognitive process and emotion regulation strategy characterized by repetitive, passive focus on negative emotions and their causes and consequences. It has been consistently associated with various psychological disorders and is closely associated with internet addiction (27). Grounded in escape theory, which posits that individuals actively shift their attention to activities offering immediate gratification to avoid distress stemming from negative self-awareness, internet use represents one example of such an activity (28). To circumvent the negative emotions elicited by rumination, individuals may immerse themselves in the richly stimulating online environment. While providing short-term relief, this coping mechanism harbors latent risks—it establishes a pattern of positive reinforcement that incrementally increases the frequency and intensity of internet use, ultimately intensifying dependency (29).

Longitudinal evidence further substantiates this pathway, indicating a significant positive correlation between heightened levels of ruminative thinking and the adoption of negative, avoidant coping strategies. This suggests that rumination impairs an individual's capacity to effectively address real-world problems, fostering a predisposition to evade challenges and consequently seek emotional solace within virtual space (30, 31). A parallel mechanism is observed in research on smartphone addiction, where rumination significantly predicts susceptibility to smartphone addiction among adolescents (32), a condition that shares highly similar etiological pathways with internet addiction (33).

While existing research has validated the unidirectional predictive role of rumination on internet addiction, the question of whether and how internet addiction might reciprocally influence rumination remains poorly understood. According to the reinforcement cycle perspective inherent to escape theory, the prolonged reliance on the internet as a means of emotional escape may erode an individual's real-world emotion regulation capacities. This could lead to the entrenchment and exacerbation of ruminative cognitions due to a lack of opportunities for real-life confrontation (34). Thus, a self-perpetuating, mutually aggravating closed loop may form between the two. Presently, there remains a notable paucity of sufficiently longitudinal research and mechanistic investigation into the potential bidirectional and dynamic relationship between rumination and internet addiction, highlighting an important topic for future research.

### 1.4 Theoretical integration: an I-PACE perspective on the loneliness–rumination–internet addiction triad

To move beyond isolated pairwise associations and test a unified mediation pathway, we turn to an established theoretical framework that integrates affect, cognition, and behavior longitudinally.

The Interaction of Person-Affect-Cognition-Execution (I-PACE) model provides an integrated framework for elucidating this dynamic process (35). Within this model, loneliness functions as an affective trigger that activates rumination—a repetitive, intrusive cognitive response to negative internal states. This affective-cognitive cascade depletes executive control resources and weakens behavioral inhibition, rendering internet use a potential behavioral outlet for emotional escape. Critically, the model's embedded negative reinforcement mechanism—whereby temporary relief from ruminative distress reinforces the behavior—sustains and consolidates this maladaptive pattern (36). The I-PACE model's emphasis on process dynamics and temporal sequencing provides a relevant theoretical framework for examining the core mechanisms examined in the present study.

### 1.5 Examining longitudinal associations with RI-CLPM

Prior investigations into these relationships have predominantly relied on cross-sectional designs, which are inherently limited in their capacity to elucidate the temporal precedence and causal mechanisms linking loneliness, rumination, and internet addiction (37–41). Furthermore, existing longitudinal studies have largely been confined to exploring pairwise associations, failing to simultaneously model the dynamic interplay among all three constructs across multiple time points (42, 43). A key challenge in this domain is the confounding of between-person stability (trait-like differences) with within-person fluctuations (state-like changes). The traditional Cross-Lagged Panel Model (CLPM) struggles to disentangle whether observed longitudinal associations reflect genuine reciprocal effects or are merely artifacts of stable inter-individual differences (44, 45).

To address this limitation, Hamaker et al. (46) proposed the Random Intercept Cross-Lagged Panel Model (RI-CLPM). The RI-CLPM decomposes longitudinal covariance into between-person effects (correlations among stable, trait-like components that differentiate individuals from one another) and within-person effects (temporal relations among time-varying state components that describe how an individual's deviation from their own mean on one variable predicts subsequent deviation on another) (47). Methodological consensus increasingly favors the RI-CLPM over the CLPM for estimating cross-lagged effects when the existence of stable trait factors is presumed, as it provides a more accurate depiction of intra-individual developmental dynamics (48). To date, this analytical approach has yet to be applied to disentangling the specific triadic loop among loneliness, rumination, and internet addiction.

Compared with alternative longitudinal approaches, the RI-CLPM is well suited to our research questions. The traditional CLPM conflates trait-like stability with state-like carry-over effects, rendering it incapable of determining whether a cross-lagged association reflects genuine within-person coupling or merely stable individual differences. Latent growth curve models describe overall developmental trajectories but do not capture reciprocal within-person dynamics among multiple constructs. Multilevel models can estimate within-person associations, yet in standard applications they typically do not simultaneously model reciprocal lagged effects across multiple time-varying constructs in the way a structural equation modeling framework like RI-CLPM does. In contrast, the RI-CLPM directly models reciprocal cross-lagged paths among the time-varying components of loneliness, rumination, and internet addiction, while partialling out all stable between-person variance through random intercepts. This aligns precisely with our aim to test bidirectional and mediational processes at the within-person level.

### 1.6 The present study

To address the aforementioned gaps, the present study aims to investigate the longitudinal relationships among loneliness, rumination, and internet addiction using the RI-CLPM. This approach enables us to disentangle within-person dynamic processes from stable between-person differences, thereby allowing a more precise examination of the bidirectional and mediational hypotheses. The target variables are loneliness, rumination, and internet addiction. Building upon the theoretical and empirical foundations outlined above, we propose the following four hypotheses:

H1: loneliness and internet addiction exhibit bidirectional predictive effects over time.

H2: loneliness and rumination exhibit bidirectional predictive effects over time.

H3: rumination and internet addiction exhibit bidirectional predictive effects over time.

**Figure 1:**
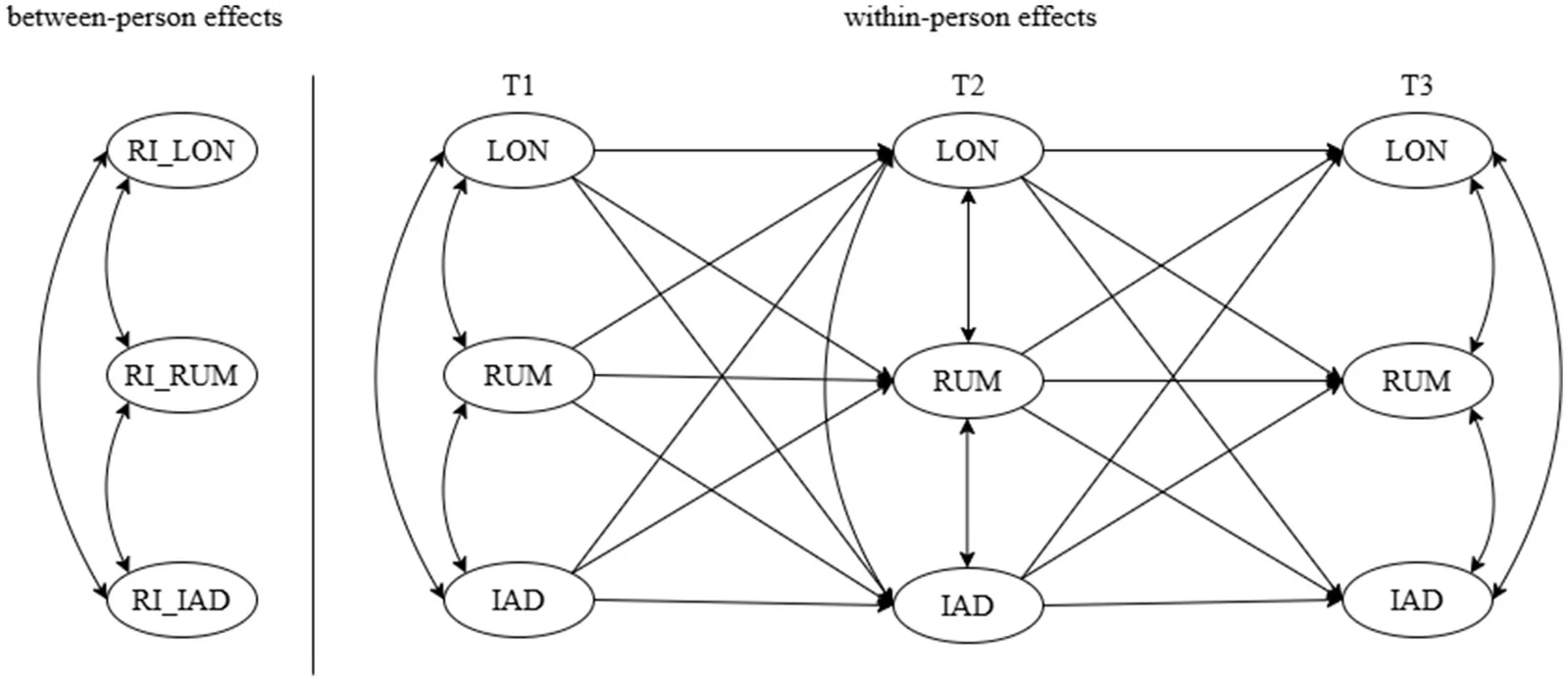
Hypothetical RI-CLPM for LON, RUM, and IAD. LON, loneliness; RUM, rumination; IAD, internet addiction; RI, random intercept; T1, time 1; T2, time 2; T3, time 3.

H4: rumination mediates the dynamic longitudinal relationship between loneliness and internet addiction.

(A conceptual diagram illustrating these hypothesized relationships is presented in Figure 1).

By testing these hypotheses within the RI-CLPM framework, the present study makes two distinct contributions. Methodologically, it applies the RI-CLPM to separate stable between-person differences from dynamic within-person processes, facilitating a more appropriate examination of reciprocal relations than traditional CLPM. Theoretically, it tests a unified longitudinal mediation model in which rumination serves as a cognitive mechanism linking loneliness to later internet addiction, addressing the directional ambiguity left by cross-sectional research.

## 2 Method

### 2.1 Participants

This study was approved by the Research Ethics Committee of the School of Psychology, Jiangxi Normal University. Written informed consent was obtained from all participating students and their parents after the study had been explained to them and with formal permission from the school authorities. Participants were informed of their right to withdraw at any time without penalty. All procedures ensured participant confidentiality and protection from physical and psychological harm.

Participants were recruited from public junior high schools in China using a cluster sampling method. Questionnaire assessments were administered at three time points: March 2023 (T1), September 2023 (T2), and March 2024 (T3). The valid sample sizes at T1, T2, and T3 were 861, 822, and 799, respectively, with an overall attrition rate of 7.2% from T1 to T3. Participants were excluded at any wave if they were absent due to illness or personal reasons, or if their responses contained missing key items (operationally defined as having more than 20% of items missing on any of the core scales—loneliness, rumination, or internet addiction) or exhibited patterned responding. The final analytic sample comprised 743 participants (384 females; *Mage* = 13.34 years, SD = 0.847).

To examine potential systematic differences between participants who completed all three waves and those who did not, attrition analysis was conducted using independent samples *t*-tests (for continuous variables) and chi-square tests (for categorical variables). Results indicated no significant differences between the attrition group (*N* = 62) and the retained sample on T1 measures of gender (χ^2^(1) = 2.175, *p* = 0.14), age (*t* = 1.77, *p* = 0.08), loneliness (*t* = −0.070, *p* = 0.944), rumination (*t* = 0.497, *p* = 0.619), and internet addiction *(t* = 0.145, *p* = 0.885), showing no systematic attrition. Little's test of Missing Completely at Random (MCAR) (49) indicated that the missing data pattern was not completely random (χ^2^/df = 1.108, *p* = 0.000). Although Little's MCAR test was significant (*p* < 0.05), in longitudinal designs missingness often satisfies the less restrictive missing at random (MAR) assumption, where the probability of missingness may depend on observed prior wave variables but not on unobserved values. To render the MAR assumption more plausible, we included all prior wave observed scores (loneliness, rumination, and internet addiction at T1 and T2) as auxiliary variables in the model. Under MAR, Full Information Maximum Likelihood (FIML) provides unbiased parameter estimates (50, 51). Therefore, FIML was employed to handle missing data in all subsequent analyses.

### 2.2 Measures

#### 2.2.1 Loneliness

The UCLA Loneliness Scale (ULS-20), originally developed by Russell (52) and later adapted for Chinese populations by Wang et al. (53), is a unidimensional measure consisting of 20 items. Items are rated on a 4-point scale ranging from one (never) to four (always). Nine items (Nos. 1, 5, 6, 9, 10, 15, 16, 19, 20) are reverse-scored, with higher total scores indicating greater levels of loneliness. Example items include “*I feel in harmony with the people around me*” (reverse-scored) and “*I feel isolated from others*.” Extant psychometric research has validated the scale's robust reliability and validity (54). In the present study, the scale showed good internal consistency, with Cronbach's α coefficients of 0.87 (T1), 0.89 (T2), and 0.90 (T3).

#### 2.2.2 Rumination

The Ruminative Response Scale (RRS), originally developed by Nolen-Hoeksema (17) and later adapted for Chinese contexts by Han and Yang (55), includes three subscales: symptom rumination (e.g., “*I often think about how lonely I am*”), brooding (e.g., “*I often think*' *Why do I always react this way* ?”), and reflective pondering (e.g., “*I often analyze recent events to understand why I feel depressed*”). The scale consists of 22 items, ranging from one (never) to four (always), with no reverse-scored items. Previous studies have demonstrated good reliability and validity of the RRS (56). In our study, the scale showed good reliability, with Cronbach's α coefficients of 0.94 (T1), 0.94 (T2), and 0.95 (T3).

#### 2.2.3 Internet addiction

This study used the Internet Addiction Scale (IAT), originally developed by Young (11) based on the Internet Addiction Diagnostic Questionnaire and later adapted for Chinese populations by Lu et al. (57). The scale is a unidimensional measure consisting of 20 items. Example items include “*Do you find that you stay online longer than you intended ?*” and “*Do you choose to spend more time online over going out with others* ?” Items are rated on a 5-point Likert scale ranging from one (rarely) to five (always), with no reverse-scored items. Higher total scores indicate more severe self-reported Internet addiction symptoms among junior high school students. The scale has demonstrated good reliability and validity (11, 57). In the present study, the scale showed good internal consistency, with Cronbach's α coefficients of 0.92 (T1), 0.93 (T2), and 0.94 (T3).It should be noted that the IAT is a screening instrument that provides a dimensional assessment of maladaptive Internet use and does not constitute a formal clinical diagnosis.

### 2.3 Procedure

Following the acquisition of informed consent from the school, participants, and their parents, the study was conducted in three waves of group-administered sessions organized by class. All assessments took place during scheduled self-study periods, with questionnaires distributed and collected on-site at each session. The content and procedures were consistent across the three waves.

Each session was coordinated with the assistance of the homeroom teacher and overseen by a trained research assistant—a graduate student in psychology who had received standardized training and was thoroughly familiar with the assessment protocol. The research assistant read standardized instructions aloud, explained the purpose and significance of the study, emphasized confidentiality and the absence of right or wrong answers, and stressed the importance of independent responding. The assistant also addressed any questions regarding the questionnaires and monitored the overall quality of the data collection process.

### 2.4 Data analysis

All statistical analyses were conducted using IBM SPSS Statistics 28.0 (IBM Corp., Armonk, NY, USA) and Mplus Version 8.3 (Muthén & Muthén, Los Angeles, CA, USA). The analytical procedure consisted of four main steps.

Step 1: Preliminary analyses. Data preprocessing, including variable transformation (reverse-coding negatively worded items and computing scores), assessment of scale reliability (Cronbach's α), testing for common method bias, descriptive statistics (means, SDs), and Pearson correlational analyses, was conducted using SPSS 28.0. To further evaluate the psychometric properties of the scales, composite reliability (CR) and average variance extracted (AVE) were calculated at each wave to assess convergent validity. Discriminant validity was examined using the heterotrait-monotrait ratio of correlations (HTMT), with values below 0.85 indicating adequate discriminant validity (58). Missing data were handled using FIML estimation in Mplus 8.3 for all subsequent models.

Step 2: Variance decomposition and measurement invariance. Intraclass correlation coefficients (ICCs) were calculated for all longitudinal variables to estimate the relative proportions of between-person vs. within-person variance across time. These ICC values indicated substantial between-person variance, justifying the use of random intercepts in the RI-CLPM. Subsequently, longitudinal measurement invariance of the instruments across the three time points was tested to ensure the stability of the measurement structure.

Step 3: RI-CLPM specification and model comparison. A random intercept cross-lagged panel model (RI-CLPM) was specified and estimated using Mplus 8.3. This model disentangles between-person from within-person effects, enabling the examination of the dynamic interplay among variables and the longitudinal mediating role of rumination. Model comparison followed a stepwise constraint strategy: an unconstrained baseline model was first established, followed by a series of increasingly constrained models in which autoregressive and cross-lagged paths were progressively equated over time. Model fit was evaluated using the CFI, RMSEA, and SRMR. Adequate model fit was indicated by CFI values close to or exceeding 0.90 (59), and RMSEA and SRMR values below 0.08 and 0.10, respectively (60). For model comparisons, changes in CFI and RMSEA were examined; a ΔCFI not exceeding 0.01 and a ΔRMSEA not exceeding 0.015 were considered indicative of acceptable fit for the constrained model i.e., supporting equality constraints across time (61).

Step 4: Mediation testing. Age and gender were included as predictors of the random intercepts to control for the influence of these time-invariant covariates on individuals' stable trait levels. The longitudinal mediating effect of rumination in the relationship between loneliness and internet addiction was tested using the bootstrap method in Mplus 8.3. A significant mediation effect was established if the 95% bias-corrected confidence interval for the indirect effect did not include zero (62).

## 3 Results

### 3.1 Common method bias test

To reduce the potential threat of common method bias (CMB) inherent in self-reported data, we implemented procedural remedies (e.g., varying the wording of instructions across the three waves). Following methodological recommendations (63, 64), CMB was assessed by comparing a single-factor and multi-factor comparison method, in which all items loaded onto one latent factor, with the theoretical multi-factor measurement model for each wave. As shown in Table 1, the multi-factor model demonstrated substantially better fit than the single-factor model at all three waves (all Δχ^2^ > 4,000, Δ*df* = 3, *p* < 0.001). These results indicate that a single common factor cannot adequately account for the item covariances; thus, CMB is unlikely to pose a serious threat to the validity of the study findings.

**Table 1:** Model comparison tests for common method bias.

Stage	Model	*χ^2^*	df	Δ*χ^2^*	Δdf	*p*
T1	Single-factor1	10,681.971	1,829	4,005.837	3	< .001^***^
	Multi-factor1	6,676.134	1,826			
T2	Single-factor2	11,851.808	1,829	4,193.743	3	< .001^***^
	Multi-factor2	7,658.065	1,826			
T3	Single-factor3	12,029.491	1,829	4,754.924	3	< .001^***^
	Multi-factor3	7,274.567	1,826			

^*^p < 0.05, ^**^p < 0.01, ^***^p < 0.001.

To complement the single-factor test, we further employed the unmeasured latent method construct (ULMC) approach. Specifically, a common method factor was added to the theoretical measurement model and constrained to be orthogonal to all substantive latent factors. The inclusion of the latent method factor led to improved model fit (ΔCFI = 0.040, ΔTLI = 0.040, ΔRMSEA = −0.004, and ΔSRMR = −0.016).

Taken together, the convergence of evidence from both the single-factor test and the ULMC analysis suggests that common method variance is unlikely to account for the observed associations among the study variables or to pose a substantial threat to the interpretation of the substantive findings.

### 3.2 Descriptive statistics and correlation analyses

Descriptive statistics and correlation matrices for loneliness, rumination, and internet addiction are presented in Table 2. The results revealed that all three variables were significantly and positively correlated with each other across the three assessment waves (0.318 ≤ *r* ≤ 0.775, ^**^p < 0.01). This indicates significant concurrent and sequential associations among the constructs, satisfying the preliminary assumptions for cross-lagged analysis.

**Table 2:** Bivariate correlations between main variables at three measurement points.

Variable	M ±SD	CR	AVE	1	2	3	4	5	6	7	8	9	10
1. Age	13.34 ± 0.847	–	–	1									
2. T1 loneliness	2.092± 0.503	0.876	0.502	0.093^*^	1								
3. T1 rumination	1.908± 0.608	0.917	0.501	−0.103^**^	0.486^***^	1							
4. T1 internet addiction	1.876 ± 0.722	0.875	0.501	0.085^*^	0.446^***^	0.537^***^	1						
5. T2 loneliness	2.101 ± 0.520	0.895	0.549	0.104^**^	0.638^***^	0.453^***^	0.359^***^	1					
6. T2 rumination	1.894 ± 0.617	0.925	0.53	−0.057	0.438^***^	0.688^***^	0.444^***^	0.608^***^	1				
7. T2 internet addiction	1.937± 0.757	0.889	0.533	0.078^*^	0.431^***^	0.450^***^	0.721^***^	0.474^***^	0.576^***^	1			
8. T3 loneliness	2.117 ± 0.537	0.913	0.599	0.094^*^	0.585^***^	0.396^***^	0.318^***^	0.725^***^	0.532^***^	0.449^***^	1		
9. T3 rumination	1.923 ± 0.600	0.924	0.527	−0.041	0.383^***^	0.574^***^	0.356^***^	0.500^***^	0.720^***^	0.486^***^	0.605^***^	1	
10. T3 internet addiction	1.959 ± 0.753	0.891	0.54	0.090^*^	0.369^***^	0.379^***^	0.632^***^	0.427^***^	0.491^***^	0.775^***^	0.480^***^	0.556^***^	1

M, mean; SD, standard deviation; CR, composite reliability; AVE, average variance extracted; T1, Time 1; T2, Time 2; T3, Time 3; *N* = 743. Pearson correlation coefficients are reported below the diagonal. ^*^p < 0.05, ^**^p < 0.01, ^***^p < 0.001.

To justify the use of the random-intercept cross-lagged panel model (RI-CLPM), a two-level random-intercept null model was estimated to partition the total variance into between-person and within-person components. The estimated between-person (random intercept) variance and within-person (residual) variance were 0.209 and 0.202 for loneliness, 0.177 and 0.212 for rumination, and 0.213 and 0.203 for self-reported Internet addiction, respectively. The corresponding intraclass correlation coefficients (ICCs), derived from these variance components, were 0.509 for loneliness, 0.455 for rumination, and 0.512 for self-reported Internet addiction. These findings indicate that 50.9%, 45.5%, and 51.2% of the total variance in loneliness, rumination, and self-reported Internet addiction, respectively, reflected stable between-person (trait-like) differences, whereas the remaining variance reflected within-person fluctuations over time. Together, these results demonstrate substantial variability at both the between-person and within-person levels, thereby supporting the use of the RI-CLPM. The detailed variance components are additionally reported in Supplementary Table S1.

Across the three waves, composite reliability values ranged from.876 to.913 for loneliness, 0.917 to 0.925 for rumination, and 0.875 to 0.891 for internet addiction, all above the recommended 0.70 threshold. The AVE values ranged from 0.501 to 0.599, exceeding the 0.50 criterion, thereby supporting convergent validity. Discriminant validity was examined using the HTMT criterion; all HTMT values between construct pairs were below 0.85, with loneliness-rumination HTMTs ranging from 0.452 to 0.747, loneliness-internet addiction HTMTs from 0.283 to 0.485, and rumination-internet addiction HTMTs from 0.301 to 0.554, indicating that the three constructs are empirically distinct.

### 3.3 Longitudinal measurement invariance testing

Four nested models representing increasing levels of longitudinal measurement invariance were tested for each key construct across the three time points: configural invariance (M1), weak invariance (M2; equal factor loadings), strong invariance (M3; equal item intercepts), and strict invariance (M4; equal residual variances). The change of CFI (ΔCFI) (60) was a stable indicator for model comparison, and if ΔCFI ≤ 0.01 and ΔRMSEA ≤ 0.015, the models would be determined to be invariant.

As shown in Table 3, all models from M1 to M3 demonstrated acceptable fit, and changes from M2 to M3 fell well within the recommended cutoffs for all three scales (all ΔCFI ≤ 0.001, ΔRMSEA ≤ 0.005), confirming strong (scalar) invariance. For strict invariance (M4), the loneliness scale failed to meet the criteria (ΔCFI = −0.010, ΔRMSEA = 0.028). In contrast, both the rumination scale (ΔCFI = −0.002, ΔRMSEA = 0.008) and the Internet addiction scale (ΔCFI = 0.000, ΔRMSEA = −0.001) met strict invariance criteria.

**Table 3:** Longitudinal measurement invariance tests for each scale.

Variable	Model	χ^2^	df	CFI	TLI	SRMR	RMSEA	AIC	BIC	aBIC	Model comparison	ΔCFI	ΔRMSEA
Loneliness	M1	39.142	39	1.000	1.000	0.013	0.002	8,417.699	8,652.844	8,490.901	—	—	—
	M2	46.442	44	1.000	0.999	0.022	0.009	8,414.999	8,627.091	8,481.024	M2-M1	0.000	0.007
	M3	59.193	52	0.999	0.999	0.024	0.014	8,411.750	8,586.957	8,466.293	M3-M2	−0.001	0.005
	M4	138.476	60	0.989	0.988	0.045	0.042	8,475.033	8,613.354	8,518.093	M4-M3	−0.010	0.028
Rumination	M1	25.116	15	0.999	0.997	0.010	0.030	5,203.195	5,383.012	5,259.173	—	—	—
	M2	28.283	21	0.999	0.998	0.017	0.022	5,194.362	5,346.515	5,241.728	M2-M1	0.000	−0.008
	M3	40.120	27	0.998	0.998	0.021	0.026	5,194.199	5,318.687	5,232.953	M3-M2	−0.001	0.004
	M4	61.630	33	0.996	0.996	0.021	0.034	5,203.709	5,300.534	5,233.851	M4-M3	−0.002	0.008
Internet addiction	M1	95.931	39	0.994	0.990	0.015	0.044	11,914.007	12,149.153	11,987.209	—	—	—
	M2	101.805	44	0.994	0.991	0.018	0.042	11,909.881	12,121.973	11,975.907	M2-M1	0.000	−0.002
	M3	121.986	52	0.993	0.991	0.026	0.043	11,914.063	12,089.269	11,968.605	M3-M2	−0.001	0.001
	M4	123.878	54	0.993	0.991	0.026	0.042	11,913.305	12,083.901	11,966.413	M4-M3	0.000	−0.001

M1, configural invariance model; M2, metric invariance model; M3, scalar invariance model; df, degrees of freedom; CFI, comparative fit index; TLI, Tucker-Lewis index; SRMR, standardized root mean square residual; RMSEA, root mean square error of approximation; AIC, Akaike information criterion; BIC, Bayesian information criterion; aBIC, sample-size adjusted Bayesian information criterion.

Consistent with Putnick and Bornstein (65), scalar (strong) invariance—rather than strict (residual) invariance—is generally considered necessary and sufficient for meaningful comparisons of latent means across time or groups; strict invariance imposes additional constraints that are often deemed unnecessarily strict for testing common factor hypotheses. Although the loneliness scale did not meet the criteria for strict invariance, all three constructs achieved strong invariance, ensuring that the measurement structures were comparable across time points. Establishing strong (scalar) invariance indicates that the latent constructs carry the same meaning and have equivalent measurement parameters across the three waves. This is a critical prerequisite for the RI-CLPM, because it ensures that any observed changes in the within-person relations among loneliness, rumination, and internet addiction reflect genuine shifts in the underlying psychological processes, rather than changes in how the constructs were measured. In other words, the temporal dynamics captured by the cross-lagged paths are not artifacts of measurement non-equivalence, thereby strengthening the validity of our inferences about developmental processes.

### 3.4 RI-CLPM analysis

To examine the dynamic relationships among loneliness, rumination, and internet addiction, a RI-CLPM across three time points was constructed. A baseline model (M1) was first specified, in which all autoregressive and cross-lagged paths were freely estimated. Subsequently, three nested constrained models were established sequentially: M2 constrained the autoregressive paths to be equal across adjacent time points; M3 constrained the cross-lagged paths to be equal over time; and M4 simultaneously constrained both the autoregressive and cross-lagged paths to be equal across time.

Model comparison results indicated that all models demonstrated acceptable fit (ΔCFI < 0.01). Considering the overall pattern of fit indices, M4 exhibited the most optimal model fit (χ^2^ = 28.547, df = 24, CFI = 0.999, TLI = 0.998, SRMR = 0.017, RMSEA = 0.016; see Table 4 for details). Furthermore, its constraint specifications enhanced the theoretical interpretability of the results while maintaining model parsimony. Therefore, M4, which included age and gender as time-invariant covariates, was selected as the final analytical model for testing the mediating role of rumination in the relationship between loneliness and internet addiction.

**Table 4:** Model fit and model comparisons for the RI-CLPM.

Model	χ^2^	df	CFI	TLI	SRMR	RMSEA	AIC	BIC	aBIC	Model comparison	ΔRMSEA	ΔCFI
M1	20.180	15	0.999	0.996	0.015	0.022	16,758.548	17,044.411	16,847.538	–	–	–
M2	23.444	18	0.999	0.996	0.016	0.020	16,755.811	17,027.842	16,840.495	M2-M1	−0.002	0.000
M3	24.040	21	0.999	0.998	0.016	0.014	16,750.407	17,008.606	16,830.786	M3-M1	−0.008	0.000
M4	28.547	24	0.999	0.998	0.017	0.016	16,748.914	16,993.281	16,824.986	M4-M1	−0.006	0.000

df, degree of freedom; CFI, comparative fit index; TLI, Tucker-Lewis index; SRMR, standardized root mean square residual; RMSEA, root mean square error of approximation; AIC, Akaike information criterion; BIC, Bayesian information criterion; aBIC, sample-size adjusted Bayesian information criterion; M1, unconstrained model; M2, autoregressive paths fixed to be time-invariant; M3, cross-lagged paths fixed to be time-invariant; M4, autoregressive and cross-lagged paths fixed to be time-invariant.

**Figure 2:**
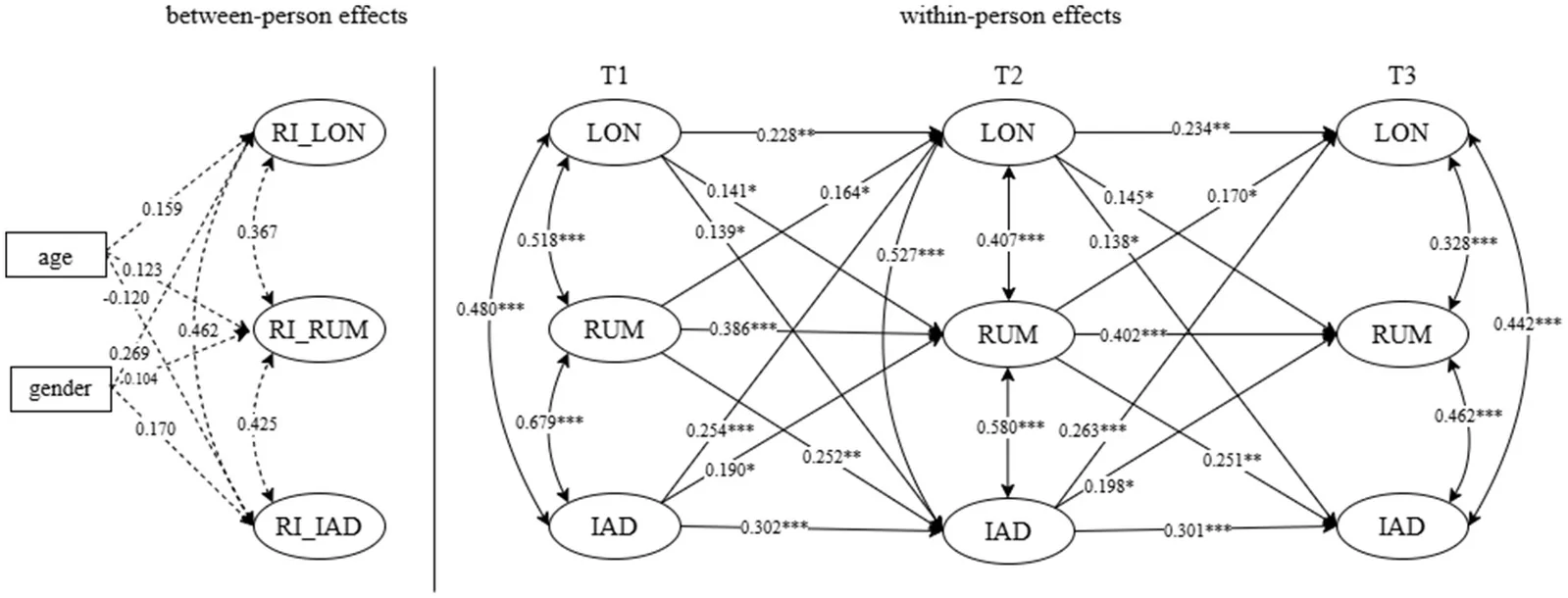
The RI-CLPM with age and gender as covariates. Coefficients in the figure represent standardized path estimates; solid lines indicate significant paths, whereas dashed lines indicate nonsignificant paths; Between-person level and Within-person level refer to the between- and within-person levels of analysis, respectively; RI, random intercept, LON, loneliness, RUM, rumination, IAD, internet addiction. *p < 0.05, **p < 0.01, ***p < 0.001.

Figure 2 displays the corresponding path diagram with standardized coefficients.

Between-person effects:

Results from the RI-CLPM (see Figure 2) indicated that, after controlling for gender, age, and within-person fluctuations, the correlations among the random intercepts (i.e., the stable between-person components) of loneliness, rumination, and internet addiction were not statistically significant (all *p* > 0.05).

Within-person effects:

(1) Autoregressive paths

All variables exhibited significant within-person autoregressive effects, indicating that deviations from an individual's own typical psychological state tended to persist across successive time points: loneliness, β*s* = 0.228–0.234, *p* < 0.01; rumination, β*s* = 0.386–0.402, *p* ≤ 0.001; and internet addiction, β*s* = 0.301–0.302, *p* < 0.001.

(2) Cross-lagged paths

Predictive Effects of Loneliness: T1 loneliness significantly and positively predicted T2 rumination (β = 0.141, *p* = 0.025) and T2 Internet addiction symptoms (β = 0.139, *p* = 0.015); T2 loneliness significantly and positively predicted T3 rumination (β = 0.145, *p* = 0.022) and T3 Internet addiction symptoms (β = 0.138, *p* = 0.015).

Predictive Effects of Rumination: T1 rumination significantly and positively predicted T2 loneliness (β = 0.164, *p* = 0.043) and T2 Internet addiction symptoms (β = 0.252, *p* = 0.003). T2 rumination significantly and positively predicted T3 loneliness (β = 0.170, *p* = 0.041) and T3 Internet addiction symptoms (β = 0.251, *p* = 0.003).

Predictive Effects of Internet Addiction: T1 Internet addiction symptoms significantly and positively predicted T2 loneliness (β = 0.254, *p* < 0.001) and T2 rumination (β = 0.190, *p* = 0.018). T2 Internet addiction symptoms significantly and positively predicted T3 loneliness (β = 0.263, *p* < 0.001) and T3 rumination (β = 0.198, *p* = 0.014).

(3) Longitudinal mediating role of rumination

The nonparametric percentile bootstrap analysis revealed a statistically significant indirect effect of T2 rumination between T1 loneliness and T3 Internet addiction symptoms, although the standardized indirect effect was modest (β = 0.037, 95% CI [0.000, 0.073]). This finding provides preliminary support for the longitudinal mediating role of rumination in the relationship between loneliness and subsequent Internet addiction. In contrast, the indirect pathway from T1 Internet addiction to T3 loneliness through T2 rumination was not significant (β = 0.030, 95% CI [−0.006, 0.067]). These findings suggest a unidirectional mediation pattern. Detailed statistics are presented in Table 5.

**Table 5:** Longitudinal mediation effect of rumination at the within-person level.

Path	β	Mediation effect	95% CI	*p*
T1 Loneliness-T2 Rumination-T3 Internet Addiction	0.035	0.037	[0.000, 0.073]	0.047
T1 Internet Addiction-T2 Rumination-T3 Loneliness	0.032	0.030	[−0.006, 0.067]	0.101

The ‘β' column reports the standardized indirect effect estimate for each specific pathway. Because the model contains only one mediator per pathway, the specific indirect effect equals the total indirect effect; the “Mediation” column provides this same estimate for clarity of presentation. Bias-corrected bootstrap confidence intervals are based on 2,000 resamples. Covariates (age, gender) were controlled at the between-person level. T1, Time 1; T2, Time 2; T3, Time 3. Statistical significance was indicated by confidence intervals excluding zero (*p* < 0.05).

## 4 Discussion

This study employed the RI-CLPM to analyze three-wave longitudinal data, systematically examining the dynamic relationships among loneliness, rumination, and self-reported Internet addiction symptoms in junior high school students. A methodological advantage of this approach lies in its ability to disentangle stable between-person differences from within-person temporal fluctuations. The findings provide preliminary longitudinal support for rumination as one potential within-person pathway linking loneliness to subsequent self-reported Internet addiction symptoms and suggest that this indirect pathway primarily operates from loneliness to Internet addiction rather than in the opposite direction. These findings contribute to a more nuanced understanding of the within-person processes underlying adolescent self-reported Internet addiction symptoms and extend current theoretical perspectives by distinguishing stable trait-level differences from dynamic within-person developmental processes.

### 4.1 Between-person effects of loneliness, rumination, and internet addiction

At the between-person level, the correlations among the random intercepts for loneliness, rumination, and Internet addiction were not statistically significant. Within the RI-CLPM framework, random intercepts capture individuals' relatively stable, trait-like propensities over time (20). This result indicates that, when considered as enduring dispositions, a heightened tendency toward loneliness, habitual rumination, or susceptibility to Internet addiction symptoms does not inherently coincide with elevated levels of the other two traits. Stated differently, these three constructs appear to be relatively independent at the trait level, likely underpinned by distinct psychological foundations.

The discrepancy between the substantial observed correlations in Table 2 and the non-significant random intercept correlations warrants further explanation. Observed correlations in Table 2 reflect an amalgamation of stable between-person differences and time-specific within-person fluctuations, as well as shared occasion-specific variance (e.g., transient mood states, common method variance from simultaneous measurement at each wave). When the RI-CLPM decomposes this covariance, the random intercept captures only the trait-like, time-invariant component of each construct. The non-significant random intercept correlations therefore suggest that the stable, trait-like propensity toward loneliness, habitual rumination, or problematic Internet use susceptibility does not necessarily co-occur with elevated trait levels of the other two constructs. In other words, the moderate to strong observed correlations may largely reflect shared within-person dynamics and occasion-specific factors rather than overlapping trait dispositions. This illustrates the value of the RI-CLPM in isolating the locus of these associations. This conceptual distinction is consistent with the distinction between between-person and within-person variance articulated by Hamaker et al. (46) and further elaborated by Mulder and Hamaker (66).

This perspective diverges from the view often implied in cross-sectional research, which may treat these variables as components of a shared trait cluster (67, 68). Such designs are limited in their ability to disentangle stable trait-level associations from transient, state-like co-variation (69). By applying the RI-CLPM, the present study explicitly distinguishes between these levels of analysis, thereby offering a refined conceptual understanding and providing a basis for developing differentiated interventions tailored to specific trait predispositions.

### 4.2 Within-person effects of loneliness, rumination, and internet addiction

At the within-person level, loneliness at a given time point significantly predicted subsequent increases in both rumination and self-reported Internet addiction; rumination, in turn, predicted later elevations in self-reported Internet addiction and loneliness; and self-reported Internet addiction positively forecasted future loneliness and rumination. The finding is consistent with prior research (15, 23, 70). These dynamic interrelations provide a process-oriented framework for understanding the co-occurrence and mutual exacerbation of mental health and behavioral problems in adolescence.

First, the observed pathway from loneliness to rumination supports the conceptualization of loneliness as a chronic social stressor (71, 72). When individuals perceive a deficit in social connectedness, they become increasingly susceptible to repetitive, negative thinking about their circumstances and emotional states. This ruminative cognitive style, rather than facilitating resolution, amplifies the distressing experience of loneliness, thereby engendering a self-perpetuating cognitive–emotional vicious cycle.

Second, the pathways from rumination to problematic Internet use and from problematic Internet use to loneliness are consistent with a compensatory cycle model of escape–reinforcement. Consistent with the Compensatory Internet Use Theory (73), the emotional distress inherent to rumination drives individuals to seek refuge in the online world as a digital sanctuary from real-life challenges, attaining immediate gratification and a sense of belonging through online social interactions and gaming. However, excessive immersion in virtual environments displaces the time and effort necessary for cultivating real-world social competencies.

Moreover, the often superficial and utilitarian nature of online social exchanges may deepen individuals' disillusionment with and estrangement from offline interpersonal relationships, thereby intensifying their fundamental experience of loneliness (74–76). By leveraging longitudinal data, the present study furnishes temporal evidence for this self-reinforcing cycle, indicating that loneliness, rumination, and Internet addiction symptoms do not merely coexist but rather constitute a dynamically coupled cluster of risk factors that mutually catalyze one another across time.

### 4.3 Reciprocal within-person dynamics between loneliness and internet addiction

The core analyses of this study confirmed the longitudinal mediating role of rumination, yet this effect demonstrated clear directionality. Bootstrap analyses revealed that the indirect pathway from within-person loneliness to self-reported Internet addiction via heightened rumination was significant, aligning with prior cross-sectional findings (67).

At the within-person level of fluctuation, when individuals felt lonelier than usual (i.e., increased intraindividual loneliness), they exhibited more pronounced rumination at the subsequent time point; this elevated rumination, in turn, predicted a higher tendency toward Internet addiction symptoms at the following wave. These findings suggest that loneliness confers risk for subsequent problematic Internet use through the mediating pathway of rumination. This pattern is consistent with prior evidence indicating that adolescents experiencing heightened loneliness are more inclined to adopt maladaptive coping strategies and become entrenched in ruminative thinking about negative affect (70, 77). Rumination depletes self-regulatory resources and reinforces negative self-perceptions (24), thereby driving individuals to seek refuge in the online world as a safe haven for escaping aversive cognitive experiences and fulfilling belongingness needs (78). By advancing cross-sectional correlations (27, 31) to the level of temporal inference, the present study identifies rumination as a critical juncture through which intraindividual emotional distress becomes translated into externalizing problem behaviors amidst fluctuations in psychological states.

From the perspective of cognitive-behavioral theory (CBT), maladaptive cognitions constitute a core maintaining factor in emotional and behavioral disorders (79). Loneliness, as a distressing and persistent negative affective state, is not a static experience; rather, it activates and consolidates rumination—a passive, repetitive focus on negative emotions and their causes and consequences (78). From a social-cognitive standpoint, individuals experiencing chronic loneliness tend to develop heightened vigilance for social threat and negative self-schemas regarding the social world (71). When such individuals perceive social deficits or interpersonal threats, these schemas drive them to dwell repetitively on their own inadequacies, the causes of social failures, and the prospect of future isolation—thereby ensnaring them in a vicious cycle between loneliness and rumination.

Rumination is not affectively neutral; it consumes psychological resources and intensifies negative emotional states, thereby creating conditions that may facilitate the development of Internet addiction symptoms. According to escape theory, individuals are motivated to withdraw from aversive self-awareness and distressing emotional states (e.g., the persistent psychological discomfort induced by rumination) by engaging in activities that absorb attention and temporarily suspend self-reflection (28). The online environment, characterized by its immersive nature and low cognitive demands, provides a readily accessible means of temporary emotional escape. Consequently, the psychological distress associated with rumination may increase the tendency to engage in excessive Internet use as an avoidant coping strategy.

Furthermore, our findings are consistent with the integrative I-PACE framework (35), which conceptualizes Internet addiction as the result of dynamic interactions among affective, cognitive, and behavioral processes. Specifically, loneliness may function as an affective trigger that promotes rumination as a maladaptive cognitive response. This persistent cognitive preoccupation may gradually deplete self-regulatory resources, intensify emotional distress, and increase reliance on Internet use as a means of emotional escape, thereby increasing the likelihood of developing Internet addiction symptoms over time (70, 80). The present findings therefore provide longitudinal support for one potential within-person pathway proposed by the I-PACE model.

In contrast, the indirect pathway through which self-reported Internet addiction contributed to subsequent loneliness via rumination was not statistically significant. This finding suggests that the mechanisms through which problematic Internet use contributes to loneliness may be more direct or may operate through alternative mediating processes, such as reduced offline social interaction, impaired sleep quality, academic frustration, or deterioration in interpersonal functioning. Rather than necessarily increasing ruminative thinking, excessive Internet use may contribute to loneliness primarily through behavioral displacement and reduced opportunities for meaningful face-to-face social engagement. This directional asymmetry suggests that rumination may play a more prominent role in translating loneliness into Internet addiction symptoms than in explaining the reverse pathway. Future theoretical models may therefore benefit from distinguishing the mechanisms underlying these two opposing longitudinal processes.

Although the indirect effect reached statistical significance, its standardized magnitude was modest (β = 0.037, 95% CI [0.000, 0.073]), and the lower bound of the confidence interval approached zero. Accordingly, these mediation findings should be interpreted with appropriate caution. Rather than providing conclusive evidence for a mediating mechanism, the present results offer preliminary longitudinal support for the possibility that rumination may represent one within-person cognitive pathway linking loneliness to subsequent self-reported Internet addiction symptoms. The modest effect size also indicates that rumination explains only a limited proportion of the longitudinal association between loneliness and Internet addiction symptoms. Other cognitive, emotional, behavioral, and contextual mechanisms—including maladaptive coping strategies, emotion regulation difficulties, offline social withdrawal, family environment, and peer relationships—may also contribute to this developmental process. Therefore, future research should incorporate additional theoretically relevant mediators, employ larger and more diverse longitudinal samples, and include more measurement occasions to evaluate the robustness, generalizability, and practical significance of this indirect pathway. Moreover, because the RI-CLPM is based on observational longitudinal data, the present findings should not be interpreted as establishing definitive causal mechanisms. Experimental, quasi-experimental, and intervention studies are needed to further evaluate the causal role of rumination in the development of Internet addiction symptoms.

Overall, the present findings have important methodological and theoretical implications. Methodologically, the present study extends previous longitudinal research by employing the random intercept cross-lagged panel model (RI-CLPM) to distinguish stable between-person differences from within-person developmental processes, thereby providing a more precise examination of the longitudinal associations among loneliness, rumination, and self-reported Internet addiction symptoms. By separating these two sources of variance, the RI-CLPM reduces the risk of conflating stable individual differences with within-person change, thereby enabling a more accurate characterization of longitudinal developmental processes. Theoretically, the present findings refine current conceptualizations of the reciprocal longitudinal association between loneliness and self-reported Internet addiction symptoms by demonstrating that the underlying within-person processes are direction-specific rather than symmetrical. These findings highlight the importance of distinguishing direction-specific within-person processes in future theoretical development and longitudinal research.

## 5 Study limitations

Despite its contributions, the present study has several limitations that warrant cautious interpretation. First, all core variables were assessed using self-report questionnaires, which may introduce biases related to self-perception, social desirability, and memory recall. Moreover, the exclusive reliance on self-reports raises the potential risk of common method variance (CMV). While statistical analyses suggested that common method variance was unlikely to substantially influence the interpretation of the findings, future research would greatly benefit from incorporating multi-informant assessments (e.g., from parents, teachers, or peers) or objective behavioral indicators (e.g., digital media usage logs) to enhance objectivity (81). Additionally, it should be noted that the IAT is a self-report screening instrument rather than a clinical diagnostic tool; thus, the findings pertain to dimensional variations in maladaptive Internet use and should not be equated with clinical diagnoses of Internet addiction.

Second, regarding the analytical framework, although the RI-CLPM accounts for stable time-invariant individual differences, it remains an observational approach and cannot eliminate the impact of unmeasured time-varying confounding factors (e.g., dynamic environmental variables like recent life events or changes in social support). These unobserved time-varying factors may lead to residual confounding in the estimation of the cross-lagged effects.

Third, although the three-wave longitudinal design (with 6-month intervals) allowed for an examination of cross-lagged relationships, it has inherent limitations in capturing more fine-grained, short-term dynamics (e.g., daily or weekly fluctuations) within the proposed cycle. Furthermore, although three waves satisfy the minimum requirement for estimating RI-CLPMs, estimating complex reciprocal longitudinal processes with only three time points may lead to reduced precision and stability of parameter estimates. Future studies using intensive longitudinal methods (e.g., experience sampling or daily diaries) with four or more measurement waves could help uncover more precise proximal, bidirectional, or nonlinear within-person processes.

Fourth, the generalizability of these findings is constrained by the narrow age range of the sample, which consisted almost exclusively of 13-year-old early adolescents. Developmental trajectories of loneliness, rumination, and internet addiction may shift significantly across mid- and late-adolescence due to cognitive maturation and changing social contexts. Therefore, caution should be exercised when applying these conclusions to adolescents as a whole. Future research employing accelerated longitudinal designs with wider age ranges is needed to map how these bidirectional dynamics unfold across the full span of adolescence.

Fifth, although gender and age were included as covariates and showed no significant effects in the present sample, future research with larger or more diverse samples should continue to examine their potential moderating effects on the observed pathways.

## 6 Theoretical and practical implications

This study contributes to methodological and theoretical understanding of the dynamic relationships among loneliness, rumination, and internet addiction. Theoretically, previous longitudinal research has largely focused on pairwise associations among loneliness, rumination, and internet addiction. By modeling reciprocal within-person associations among all three constructs and testing the mediating role of rumination, the present study contributes to understanding the dynamic mechanisms underlying adolescent internet addiction symptoms and provides additional longitudinal evidence that may inform prevention and intervention efforts. Methodologically, whereas existing evidence has often relied on cross-sectional designs (37, 39), the present study employed a three-wave longitudinal design with RI-CLPM. This approach separates within-person from between-person variance, allowing for a more precise examination of the temporal dynamics linking loneliness, rumination, and internet addiction at the individual level.

The findings may also have implications for adolescent mental health promotion and intervention. By identifying the sequential pathway from loneliness to internet addiction through rumination, this study suggests several potential entry points for prevention and intervention. First, at the school level, schools may consider incorporating curricula or activities that facilitate self-exploration and identity integration to help adolescents derive a sense of continuity and meaning from real-world social interactions, thereby reducing the tendency to turn to the internet as an escape from loneliness. Second, with regard to rumination, positive psychology interventions—such as those focusing on enhancing positive emotional experiences, cultivating gratitude, and identifying personal strengths—may help improve emotion regulation patterns, mitigate the tendency toward repetitive negative self-focus, and weaken the mediating pathway from loneliness to internet addiction. Finally, within a family–school collaborative framework, interventions that promote supportive parent–adolescent communication and family support may help reduce internet addiction symptoms while also addressing underlying emotion regulation difficulties. By improving parent–adolescent communication and fostering supportive family environments, such approaches may help adolescents develop more adaptive coping strategies, interrupt the reciprocal cycle linking loneliness, rumination, and internet addiction symptoms, and promote a healthier balance between online and offline engagement.

## 7 Conclusion

This longitudinal study, utilizing a three-wave design and RI-CLPM, found that loneliness among junior high school students was prospectively associated with higher levels of Internet addiction symptoms through the mediating role of rumination. At the between-person level, the random intercepts of loneliness, rumination, and internet addiction were not significantly correlated; however, at the within-person level, significant bidirectional associations emerged among the three constructs. Notably, the indirect effect was observed only in the pathway from loneliness to rumination to internet addiction, and not in the reverse direction. These findings provide preliminary longitudinal evidence consistent with a cognitive mediating mechanism of rumination, addressing the inability of previous cross-sectional research to establish directionality.

It is important to emphasize that these within-person longitudinal associations are observational in nature and should not be interpreted as definitive causal mechanisms. The RI-CLPM, while offering a more refined decomposition of between- and within-person variance than traditional longitudinal models, remains an observational design that cannot rule out unmeasured time-varying confounding.

Although this study relied on self-reported data and a sample of Chinese adolescents, which may limit cross-cultural generalizability, the findings suggest the potential value of early identification and intervention targeting rumination to disrupt maladaptive internet use patterns, although these implications await testing in experimental or intervention designs. Future research should incorporate objective behavioral indicators, examine contextual moderators, and conduct cross-cultural comparisons to develop more targeted and effective intervention strategies.

Methodologically, this study demonstrates the utility of the RI-CLPM in disentangling stable between-person differences from dynamic within-person processes, thereby providing a more refined understanding of the temporal dynamics linking loneliness, rumination, and internet addiction than traditional longitudinal approaches.

## Data Availability

The datasets generated and/or analyzed during the current study are not publicly available due to ethical restrictions regarding participant confidentiality and privacy. However, they are available from the corresponding author upon reasonable request.
