# Supplementary material for: From loneliness to internet addiction in adolescents: disentangling the within-person mediating role of rumination via RI-CLPM
Source: Front Public Health. 2026 Aug 24;14:1908127. doi: 10.3389/fpubh.2026.1908127 (PMC13547008; doi:10.3389/fpubh.2026.1908127)
Supplement: Supplementary file 1 [file Table_1.docx]

**TABLE S1. Between-Person and Within-Person Variance Components of the Core Variables**

| **Variable** | **Between-Person Variance (Random Intercept)** | **Within-Person Variance (Residual)** | **ICC** |
| --- | --- | --- | --- |
| **Loneliness** | 0.209 | 0.202 | .509 |
| **Rumination** | 0.177 | 0.212 | .455 |
| **Internet Addiction** | 0.213 | 0.203 | .512 |
